# Higher Tolerance of Canopy-Forming *Potamogeton crispus* Than Rosette-Forming *Vallisneria natans* to High Nitrogen Concentration as Evidenced From Experiments in 10 Ponds With Contrasting Nitrogen Levels

**DOI:** 10.3389/fpls.2018.01845

**Published:** 2018-12-13

**Authors:** Qing Yu, Hong-Zhu Wang, Chi Xu, Yan Li, Shuo-Nan Ma, Xiao-Min Liang, Erik Jeppesen, Hai-Jun Wang

**Affiliations:** ^1^State Key Laboratory of Freshwater Ecology and Biotechnology, Institute of Hydrobiology, Chinese Academy of Sciences, Wuhan, China; ^2^University of Chinese Academy of Sciences, Beijing, China; ^3^School of Life Sciences, Nanjing University, Nanjing, China; ^4^Hubei Academy of Environmental Sciences, Wuhan, China; ^5^Department of Bioscience, Arctic Research Centre, Aarhus University, Silkeborg, Denmark; ^6^Sino-Danish Centre for Education and Research, Beijing, China

**Keywords:** whole-ecosystem experiment, growth form, submersed macrophytes, ammonium, phosphorus, phytoplankton, periphyton

## Abstract

Due to excess nutrient loading, loss of submersed macrophytes is a worldwide phenomenon in shallow lakes. Phosphorus is known to contribute significantly to macrophyte recession, but the role of nitrogen has received increasing attention. Our understanding of how high nitrogen concentrations affect the growth of submersed macrophytes, particularly under natural conditions, is still limited. In this study, we conducted experiments with canopy-forming *Potamogeton crispus* in 10 ponds subjected to substantial differences in nitrogen loading (five targeted total nitrogen concentrations: control, 2, 10, 20, and 100 mg L^-1^) and compared the results with those of our earlier published experiments with rosette-forming *Vallisneria natans* performed 1 year before. Canopy-forming *P. crispus* was more tolerant than rosette-forming *V. natans* to exposure to high NH_4_ concentrations. This is probably because canopy-forming species reach the water surface where there is sufficient light for production of carbohydrates, thereby allowing the plants to partly overcome high NH_4_ stress. Both the canopy-forming *P. crispus* and the rosette-forming *V. natans* showed clear declining trends with increasing chlorophyll *a* in the water. Accordingly, shading by phytoplankton might be of key importance for the decline in submersed macrophytes in this experiment. Both experiments revealed free amino acids (FAA) to be a useful indicator of physiological stress by high ammonium but is not a reliable indicator of macrophyte growth.

## Introduction

The mechanisms behind the loss of submersed macrophytes in shallow lakes have received ample attention due to the important role that macrophytes play for maintaining healthy conditions in such ecosystems (Blindow, 1992; Jeppesen et al., 1998; Scheffer, 1998; Körner, 2002; Wang et al., 2014). In addition to excessive loading of phosphorus (P) (Scheffer, 1998; Carpenter, 2003), high nitrogen (N) loading has also been suggested to contribute importantly to the recession of macrophytes (Moss, 2001; Jeppesen et al., 2007; Moss et al., 2013). High N may cause physiological damage to the submersed macrophytes by generating oxidative stress (Wang et al., 2010; Zhang et al., 2011), disturbing the metabolism of carbon and nitrogen (Cao et al., 2009b; Gao et al., 2015; Yuan et al., 2015) and inhibiting photosynthesis (Wang et al., 2008; Su et al., 2012). High N may also affect the plants indirectly by promoting the growth of phytoplankton (Sayer et al., 2010a,b) or periphyton (Olsen et al., 2015; Zhao et al., 2016) and hence their shading effects. The effect of high N may, however, vary with the macrophyte species present (Cao et al., 2009a,b). Different key mechanisms have been identified in various studies and it has been suggested that such difference may partly reflect variation in temporal scale of the conducted experiments and timing (season). For example, toxic effects tend to play a key role in acute tests, while shading effects of algae are important in chronic tests (Cao et al., 2004; Nimptsch and Pflugmacher, 2007; Olsen et al., 2015; Zhao et al., 2016). In a long-term pond experiment carried out by Yu et al. (2015, 2017), shading by phytoplankton was identified as the main cause of declining growth for plants of *Vallisneria natans*, and high N-induced toxic stress seemed to have negative effects in both the growing season (summer and autumn) and the low-growth season (winter); however, active growth in the growing season enabled *V. natans* to partly overcome the stress.

Growth form may also influence the response of submersed macrophytes to the stress of high N loading. As to growth form, submersed macrophytes can be divided into rosette-forming species (e.g., *V. natans*), canopy-forming species (e.g., *P. crispus*), bottom-dwelling species (e.g., *Najas marina*), and erect species (e.g., *Hydrilla verticillata*) (Chambers, 1987; Chambers and Kalff, 1987). Canopy-forming and rosette-forming species are the two most common submersed macrophytes. Canopy-forming species form canopies on the water surface, whereas rosette-forming species spread under water through formation of ramets. Thus, canopy-forming species have better access to light than rosette-forming species. Low light may aggravate ammonium (NH_4_)-related physiological stress, as suggested in a 2-month tank (2 m × 1 m × 1 m) experiment (Cao et al., 2011). This is because extra energy and carbohydrates are needed for the detoxication process (Rare, 1990; Krupa, 2003) and photosynthesis is limited under low light conditions (Cao et al., 2009a, 2011; Yuan et al., 2013, 2016). Therefore, we hypothesize that canopy-forming submersed macrophytes may be more tolerant to NH_4_ stress than rosette-forming species.

The aim of this study was to explore the effects of high N concentrations on the growth of canopy-forming submersed macrophytes and compare their tolerance to high N with that of the rosette-forming species *V. natans* reported in Yu et al. (2017), which showed that leaf length and dry mass of *V. natans* in summer declined with increasing ammonium (NH_4_) concentrations. *P. crispus,* a typical canopy-forming and cosmopolitan species (Jackson and Kalff, 1993; Hudon et al., 2000), was selected and the experiment was performed 1 year before that of Yu et al. (2017). The two experiments were undertaken in the same pond system. The purposes of our study were twofold: (1) to test whether high N concentrations affect canopy-forming *Potamogeton crispus* through physiological stress or by promoting growth of phytoplankton or periphyton to form shading effects; (2) to compare the responses of canopy-forming and rosette-forming macrophytes to high N loading.

## Materials and Methods

### Study Area and Experimental System

The experiments were conducted in 10 equally sized (ca. 0.08 ha) experimental ponds (N 30°17′17″, E 114°43′45″) being constructed from a lotus pond (culturing *Nelumbo nucifera*) by dredging surface sediments rich in nutrients and organic matter and introducing sediments and water from a nearby lake, Lake Bao’an (surface area 48 km^2^, mean water depth, Z_M,_ and 1.9 m) (see Yu et al., 2015 for the initial environmental conditions of the 10 ponds). The experimental area is located in the middle Yangtze River Basin with a warm, humid subtropical climate (annual mean air temperature ca. 19°C and precipitation ca. 1030 mm). The monthly rainfall during the experiments was 125.7 mm in Yu et al. (2017) and 214.7 mm in this study (Supplementary Table 1). The mean air and water temperatures were 28.0 and 29.1°C in Yu et al. (2017) and 22.2 and 23.7°C in this study. Light intensity was 40998 and 38776 lux in this experiment and Yu et al. (2017), respectively. Mean Secchi depth (SD) in Yu et al. (2017) and this study were similar, being 52.1 and 51.9 cm, respectively.

### Experimental Treatments

A gradient of five target concentrations of total nitrogen (TN) (control, 2, 10, 20, and 100 mg L^-1^) and two duplicates were made. The target TN concentrations of the control ponds (ca. 0.5 mg L^-1^) functioned as background concentrations. In China, a nitrogen concentration of 2 mg L^-1^ TN refers to V type of water based on environmental quality standards for surface water (General Administration of Quality Supervision, Inspection and Quarantine of the People’s Republic of China [AQSIQ], 2002b), 10 mg L^-1^ NO_3_-N refers to standards for drinking water quality (Standardization Administration of the People’s Republic of China [SAC], 2006), and 20 mg L^-1^ TN refers to primary B based on the discharge standard of pollutants for municipal wastewater treatment plants (General Administration of Quality Supervision, Inspection and Quarantine of the People’s Republic of China [AQSIQ], 2002a). In part of the United States, 100 mg L^-1^ NO_3_-N refers to water quality standards for agriculture and livestock (Xia et al., 2004). Our gradient therefore covers the range observed in the real world. To maintain target concentrations, NH_4_Cl fertilizer (NH_4_Cl, ≥99.5%, Sinopharm Chemical Reagent Co., Ltd., Shanghai) was added every month relative to the difference between the measured and target concentrations. No phosphate fertilizer was added to the ponds in the experiment.

On 15 March 2013, *P. crispus* were collected from Lake Biandantang (N 30°32′, E 114°33′), a sub-area of Lake Bao’an. Similar-sized plants were selected and cut into a unified leaf length of 15 cm. The plants were then cultured in batches for 15 days in boxes (65 cm × 41 cm × 31 cm) filled with lake water. On 1 April 2013, similar-sized plants were selected and planted in plastic pots (23 cm in top diameter, 13 cm in bottom diameter, and 13 cm in height) filled with 10 cm sediments mixed with 1/3 washed sand (three plants in each pot). Sediment was taken from the experimental pond of the control treatment, with an original total nitrogen concentration of 1.93 mg g^-1^, a total phosphorus concentration of 0.67 mg g^-1^, and an organic matter concentration of 39.8 mg g^-1^. Three pots with plants were hung on bamboo racks (6 m in length, 2 m in width, and 3 m in height) at three water layers: 0.4, 0.8, and 1.2 m, and a total of nine pots were placed in each pond. Macrophytes with lower C: N ratios (N-enriched waters) may be more readily consumed by herbivorous or omnivorous fish (Dorenbosch and Bakker, 2011). Before the experiment published in Yu et al. (2015), we performed an experiment with the same treatments but without excluding fish. All the macrophytes had been removed by the fish at the beginning of the experiment. Therefore, to ensure sufficient exposure time of macrophytes to high ammonium stress, nets with a mesh size of 2 cm × 2 cm were fixed around the bamboo racks to prevent fish from entering. The experiment lasted around two and a half month, from 1 April to 12 June 2013.

### Sampling and Measurement

All plants were harvested at the end of the experiment and washed with tap water. The number of shoots (N_Shoot_) was counted and shoot height (H_Shoot_) was measured. To prevent the accumulation of NH_4_ in plant organs, macrophytes usually incorporate it into some nitrogenous compounds [mainly free amino acids (FAA)] and/or transport it out of the organs, which processes consume carbohydrate and energy. About 100 mg of fresh shoots were ground in 5 mL 10% acetic acid solution to measure the contents of FAA. The above solution was centrifuged at 10,000 *g* for 15 min. The supernatant was used to examine FAA using the ninhydrin colorimetric method with leucine as standard (Li et al., 2004).

The dry mass of shoots (DM_Shoot_) was measured with an electronic balance (0.01 g, BL-2200H, Shimadzu Corporation, Japan) after drying at 80°C (Jinghong, DHG-9071A, Shanghai) for 48 h to constant mass. Relative growth rates (RGRs, day^-1^) of the macrophytes were calculated using the formula:

$$\begin{array}{c} RGR=\left(lnWt-lnW0\right)/T \end{array}$$

where T is the experimental period, W_t_ is the shoot number/shoot height/shoot biomass at the end of the experiment, and W_0_ is the initial shoot number/shoot height/shoot biomass of the macrophytes.

There was no water flow through the ponds during the experiments. Environmental parameters were measured every 20 days. Dissolved oxygen and water temperature at 0.4, 0.8, and 1.2 m from the water surface were measured *in situ* with a YSI ProPlus (Yellow Spring Inc., United States). The water temperature difference between 0.4 m (upper layer) and 1.2 m (lower layer) was less than 1°C. Light intensity was measured by luminometer (KONICA MINOLTA, T-10, China) at the air-water interface (just above the water surface) and 0.4, 0.8, and 1.2 m below the water surface. Water samples for chemical analysis were collected at 5 randomly chosen locations within each pond by integrating the water column with a tube sampler (height 1.5 m and diameter 10 cm). Chlorophyll *a* of phytoplankton (Chl*a*_Phyt_) was extracted with 90% acetone (at 4°C for 24 h) after filtration through GF/C filters (Whatman, GE Healthcare UK Limited, Buckinghamshire, United Kingdom), and absorbance was read at 665 and 750 nm, both before and after acidification with 10% HCl using a spectrophotometer. The Chl*a*_Peri_ concentration was calculated following the equation in Zhang and Huang (1991):

$$\begin{array}{c} C=\frac{(\mathrm{Eb}-\mathrm{Ea})*R*K*Ve}{1000*\left(R-1\right)*S} \end{array}$$

where C is the concentration of Chl*a*_Peri_ in mg m^-2^; Eb is D-value of absorbance at 665 and 750 nm before acidification with HCl; Ea is *D*-value of absorbance at 665 and 750 nm after acidification with HCl; *R* = 1.7; *K* = 11.24; V_e_ is total volume of extract in mL; and S is surface area of the slides in m^2^.

Total nitrogen and total phosphorus (TP) were determined spectrophotometrically after digestion with K_2_S_2_O_8_ solution (Huang et al., 1999). Total ammonium (NH_4_) was determined with Nessler’s reagent colorimetric method (ISO 5664, 1984; ISO 7150-1, 1984). NH_3_ was calculated following the equation in Emerson et al. (1975) and Wood (1993):

$$\begin{array}{c} NH_{3}=NH_{4}-\frac{NH_{4}}{\left[1+10^{\left(pH-pKa\right)}\right]} \end{array}$$

where pKa = 0.09018 + 2729.92/(273.2 + Temp), NH_4_ is the measured concentration of total ammonium (including both NH_3_ and NH_4_^+^), pH is the measured pH of the solution, and Temp is water temperature in °C. In Yu et al. (2017) and this study, NH_3_ and NH_4_^+^ were expressed as total ammonium. Highly significant positive correlations were found between NH_3_ and NH_4_^+^ in Yu et al. (2018) (*r* = 0.90, *n* = 9, *p* < 0.001) and in this study (*r* = 0.98, *n* = 9, *p* < 0.001), reflecting that the high concentrations of NH_3_ were accompanied by high NH_4_^+^ concentrations. Consequently, the concentration of total ammonium (NH_4_) in the two experiments serves as a combined indicator of toxicity, whether due to NH_3,_ NH_4_^+^, or both. Therefore, measured NH_4_ was used to express toxicity in both Yu et al. (2017) and this study.

In order to study the possible effects of periphyton on plants, artificial substrates were used to monitor the growth of periphyton. Three glass slides were embedded into a box with an open top and bottom, which was hung within the canopy of growing plants. Every 15 days, the glass slides were gently removed from the box for laboratory measurements after which a new set of three glass slides was embedded in the macrophytes. The periphyton growing on the glass slides was gently removed by a soft brush and flushed with 25 mL distilled water followed by filtered water, after which the concentration of periphyton chlorophyll *a* (Chl*a*_Peri_) was determined via acetone extraction.

### Statistical Analyses

Median values were used for analyses to avoid deviation caused by extreme values of TN, TP, Chl*a*_Phyt_, etc. Pearson’s correlations were used to test for relationships between macrophyte variables and environmental variables. Variables that did not follow normal distributions (Shapiro-Wilk test, *p* < 0.05) were log_10_ (*x*)-transformed and these included NH_4_, Chl*a*_Phyt_, and Chl*a*_Peri_. STATISTICA 8.0 and Microsoft Excel 2013 were used for all data analyses. Plants in pond N2b were grazed by invading fish as the net fell down. We therefore only analyzed the data from the other nine treatments.

## Results

### Growing Conditions of *P. crispus*

**FIGURE 1:**
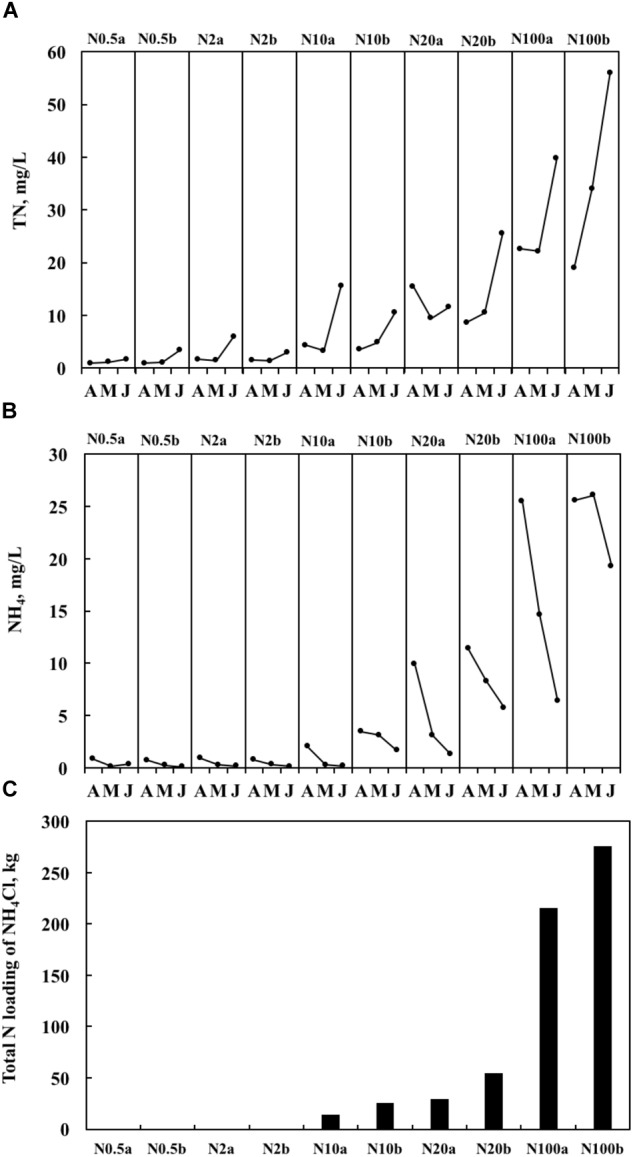
Temporal dynamics of the concentrations of total nitrogen (TN) **(A)**, the concentrations of NH_4_ (the measured concentration of total ammonium, including both NH_3_ and NH_4_^+^) **(B)**, and total nitrogen (N) loading for the entire period of the experiments **(C)** from April 2014 to June 2014. In the treatment number, the value after N indicates the target TN concentration, a and b represent two ponds with the same nitrogen treatment.

**FIGURE 2:**
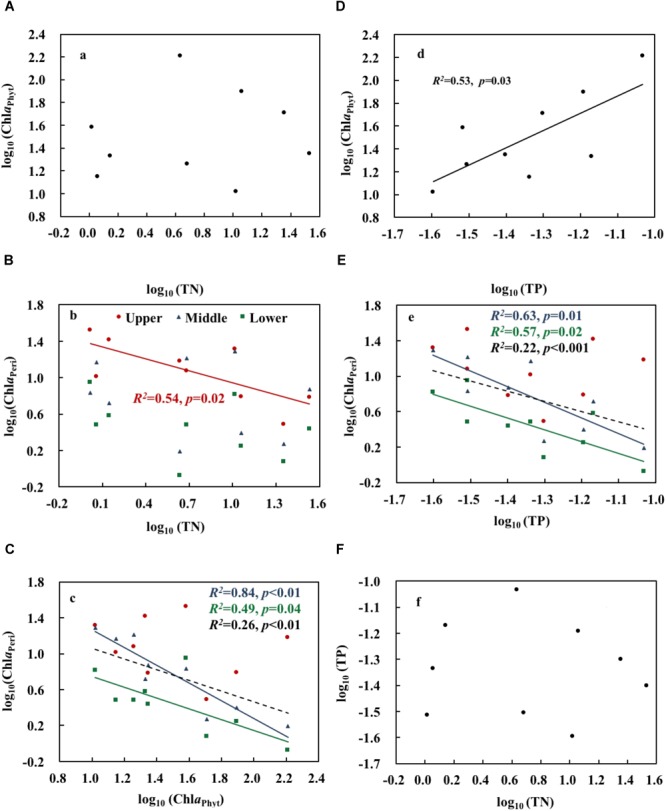
Relationships between total nitrogen, total phosphorus, phytoplankton chlorophyll a, and periphyton chlorophyll *a* **(A–F)**. Upper, middle, and lower represent the layer at 0.4, 0.8, and 1.2 m below the water surface (*n* = 9). The dotted line shows significant relationships of pooled data from all depths (*n* = 9). The red, blue, green, and black *R^2^* and *p* correspond to the red, blue, green, and black line, respectively.

Both realized TN and ammonium (NH_4_) formed a significant treatment gradient, with medians ranging from 1.0 to 34.0 mg L^-1^ (Figure 1A) and from 0.2 to 25.6 mg L^-1^ (Figure 1B), respectively. The total amounts of added fertilizer are shown in Figure 1C. Chl*a*_Phyt_ also increased significantly, with medians ranging between 10.5 and 162.4 μg L^-1^. Chl*a*_Peri_ declined from the upper (3.3–29.3 mg m^-2^) over the middle (1.8–17.0 mg m^-2^) to the lower (1.0–9.6 mg m^-2^) layers in most treatments, except N0.5a and N100b (Chl*a*_Peri_ was a little bit higher in the middle layer than the upper layer in the two treatments). Pearson’s correlations among the representative variables are given in Table 1. A highly significant positive relationship was found between TN and NH_4_ (*p* < 0.01). There was no relationship between TN and Chl*a*_Phyt_ (Figure 2A). A significant negative correlation was found between TN and Chl*a*_Peri_ in the upper layer (*p* < 0.05) (Table 1 and Figure 2B) and between Chl*a*_Peri_ and Chl*a*_Phyt_ in the middle (*p* < 0.01) and lower layers (*p* < 0.05) (Table 1 and Figure 2C). A significant positive correlation was found between TP and Chl*a*_Phyt_ (*p* < 0.05) (Figure 2D), and a negative correlation between Chl*a*_Peri_ and TP, in the middle and lower layer (*p* < 0.05) (Figure 2E). No significant correlation was found between TN and TP (Figure 2F).

**Table 1:** Pearson’s correlations coefficients (*r*-values) among environmental variables; significant correlations are shown (*p < 0.05 and **p < 0.01) in bold (*n* = 9).

		log_10_	log_10_	log_10_
	Layer	(NH_4_)	(Chl*a*_Phyt_)	(Chl*a*_Peri_)
log_10_ (TN)	Upper	0.94**	0.12	-0.64*
	Middle			–0.37
	Lower			–0.26
log_10_ (NH_4_)	Upper		–0.19	–0.68*
	Middle			–0.11
	Lower			–0.01
log_10_ (Chl*a*_Phyt_)	Upper			–0.21
	Middle			–0.86**
	Lower			–0.64*

### Growth of *P. crispus* at Contrasting Nitrogen Concentrations

**FIGURE 3:**
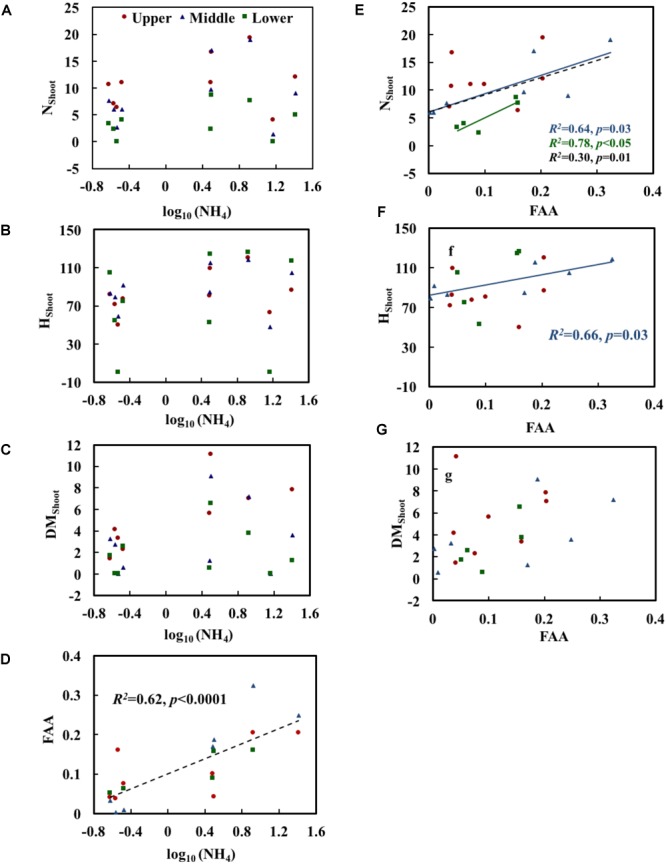
Relationships of NH_4_ (the measured concentration of total ammonium, including both NH_3_ and NH_4_^+^) with the morphological characteristics **(A–C)** and free amino acids (FAA) **(D)** of *Potamogeton crispus* and FAA **(E–G)** with morphological characteristics. Upper, middle, and lower represent the layer at 0.4, 0.8, and 1.2 m below the water surface. The dotted line shows significant relationships of pooled data from all depths (*n* = 9). The red, blue, and black *R^2^* and *p* correspond to the red, blue, and black line, respectively.

Growth and physiological variables of plants are shown in Table 2. No significant correlations were found between NH_4_ and the growth variables (Table 3). The scatterplots did not show any clear changes in growth variables with increasing NH_4_ concentrations (Figures 3A–C). FAA showed a significant increasing trend with NH_4_ when pooling data from all depths (*p* < 0.001) (Figure 3D). When regressing FAA with growth variables of plants, N_Shoot_ demonstrated a significant positive relationship with increasing FAA in the middle and lower layers (*p* < 0.05). When pooling the data from all three layers, similar results were obtained (Figure 3E). H_Shoot_ in the middle layer exhibited a significant relationship with FAA (*p* < 0.05) (Figure 3F), while DM_Shoot_ was not related to increasing FAA (Figure 3G).

**Table 2:** Characteristics of the growth and physiological variables of *Potamogeton crispus* (mean) in various experimental ponds (plants in pond N2b were grazed by invading fish because the net fell down).

Treatment	Water
number	layer	N_Shoot_	H_Shoot_	DM_Shoot_	FAA	SC
N0.5a	Upper	11	77.0	2.27	0.076	2.13
	Middle	6	91.7	0.58	0.008	1.44
	Lower	4	74.7	2.55	0.062	2.03
N0.5b	Upper	11	82.0	1.44	0.041	12.35
	Middle	8	83.0	3.24	0.032	10.74
	Lower	3	104.7	1.69	0.051	14.04
N2a	Upper	7	71.7	4.13	0.037	8.37
	Middle	6	79.3	2.73	0.002	20.99
	Lower	2	54.3	0	0	0
N10a	Upper	6	49.7	3.33	0.160	2.14
	Middle	3	58.7	0	0	0
	Lower	0	0.0	0	–	–
N10b	Upper	17	109.3	11.13	0.042	4.29
	Middle	17	115.3	9.09	0.187	5.00
	Lower	9	124.3	6.54	0.156	7.15
N20a	Upper	11	80.3	5.62	0.100	5.94
	Middle	10	84.7	1.26	0.169	6.86
	Lower	2	52.7	0.56	0.090	8.54
N20b	Upper	19	120.0	7.02	0.204	1.83
	Middle	19	118.3	7.17	0.324	3.04
	Lower	8	126	3.76	0.160	12.83
N100a	Upper	4	62.7	0	0	0
	Middle	1	48.0	0	0	0
	Lower	0	0.0	0	–	–
N100b	Upper	12	86.3	7.83	0.204	4.76
	Middle	9	104.7	3.59	0.248	6.04
	Lower	5	117.3	1.27	0	0

*N_*Shoot*_, number of shoots; H_*Shoot*_, height of shoots, cm; DM_*shoot*_, dry mass of shoots, g; FAA, free amino acids of plants, mg/g; SC, soluble carbohydrate, mg/g; “-” no sample of plants was obtained. In “treatment number” the value after N indicates the target concentration of total nitrogen, a and b represent two ponds subjected to the same nitrogen treatment. Due to its natural decay in early summer, *P. crispus* was fragile during the last measurement conducted on 12 June, preventing accurate measurements of number and height. Instead, data from 20th May (the period before the last measurement) were used in the analysis.*

**Table 3:** Pearson’s correlation coefficients (*r-*values) between growth and physiological variables of *Potamogeton crispus* relative to environmental variables (see Tables 1, 2 for explanation of the abbreviations and units).

		log_10_	log_10_	log_10_	
	Layer	(NH_4_)	(Chl*a*_Phyt_)	(Chl*a*_Peri_)	FAA
N_Shoot_	Upper	0.28	–0.65	0.34	0.26
	Middle	0.31	–0.58	**0.91^∗∗^**	**0.80^∗^**
	Lower	0.29	–**0.77^∗^**	**0.73^∗^**	**0.88^∗^**
H_Shoot_	Upper	0.40	–**0.75^∗^**	0.26	0.13
	Middle	0.26	–**0.75^∗^**	**0.89^∗∗^**	**0.81^∗^**
	Lower	0.22	–**0.79^∗^**	**0.82^∗^**	0.64
DM_Shoot_	Upper	0.39	–0.38	0.29	0.22
	Middle	0.28	–0.62	**0.81^∗^**	0.60
	Lower	0.16	–0.61	0.69	0.74

*Significant differences are shown (^∗^*p* < 0.05 and ^∗∗^*p* < 0.01) in bold (*n* = 9).*

**FIGURE 4:**
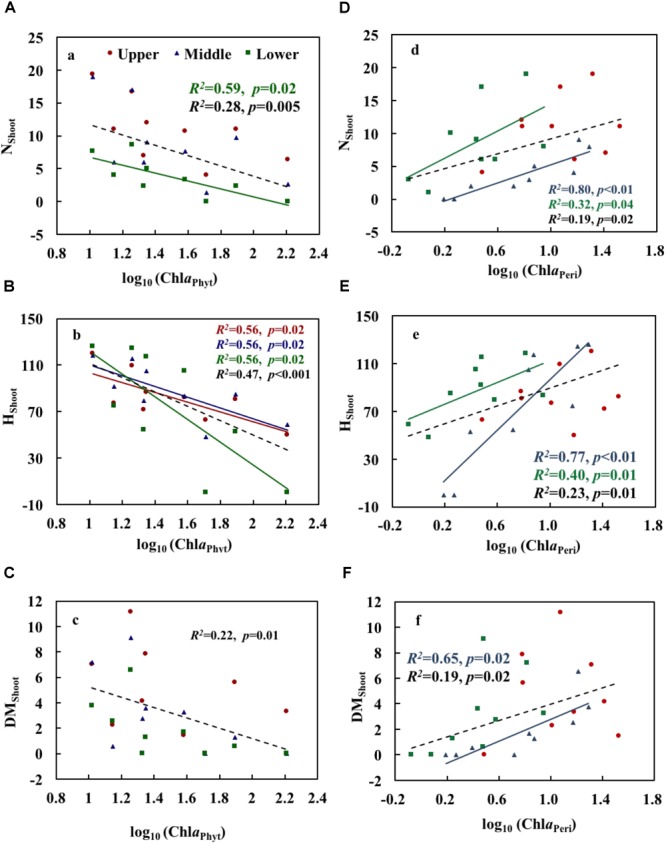
Relationships of phytoplankton chlorophyll *a* **(A–C)** and periphyton chlorophyll *a* **(D–F)** with the growth and physiological variables of *Potamogeton crispus.* Upper, middle, and lower represent the layer at 0.4, 0.8, and 1.2 m below the water surface. The dotted line shows significant relationships of pooled data from all depths (*n* = 9). The red, blue, green, and black *R^2^* and *p* correspond to the red, blue, green, and black line, respectively.

Chl*a*_Phyt_ showed significant negative correlations with N_Shoot_ and H_Shoot_ in the lower layer and with H_Shoot_ in the upper and middle layers (*p* < 0.05) (Table 3). The scatterplots also showed generally declining trends of growth variables with increasing Chl*a*_Phyt_, significantly so for N_Shoot_ in the lower layer, for H_Shoot_ in all three layers, and for all growth indices when pooling the data from all three layers (Figures 4A–C).

Chl*a*_Peri_ showed significant positive correlations with N_Shoot_ and H_Shoot_ in the middle and lower layers, and with DM_Shoot_ in the middle layer (*p* < 0.05) (Table 3). The scatterplots also demonstrated clear increasing trends of growth variables with increasing Chl*a*_Peri_, significantly so for N_Shoot_ and H_Shoot_ in the middle and lower layers, for DM_Shoot_ in the middle layer, and for all growth indices when pooling the data from all three layers (Figures 4D–F).

### Comparison Between *P. crispus* and *V. natans*

Although the precipitation varied in the experiments with *P. crispus* and *V. natans*, we maintained a fixed water level by pumping water out of the ponds after rainfall events. The average Secchi depth (SD) was similar in the two experiments, indicating that the two experiments apart from the temperature (see Discussion) were comparable despite the fact that they were conducted in two different years.

**FIGURE 5:**
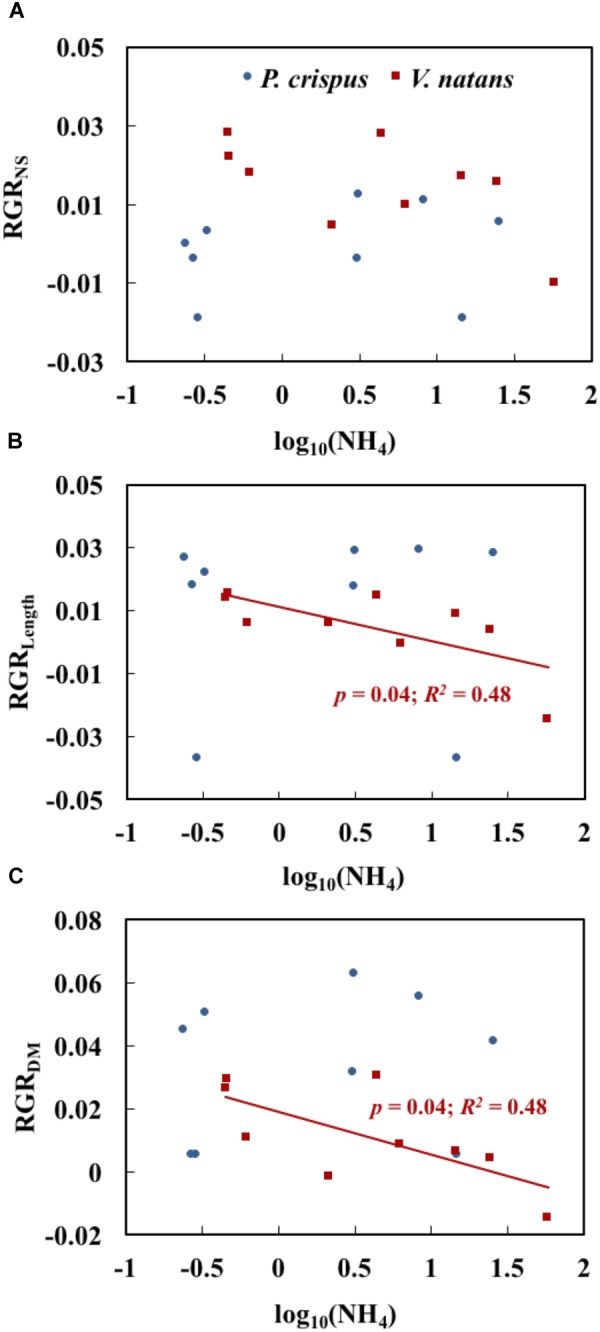
Relationships of NH_4_ (the measured concentration of total ammonium, including both NH_3_ and NH_4_^+^) with the relative growth rate of shoot number (RGR_NS_) **(A)**, relative growth rate of length (RGR_Length_) **(B)**, and relative growth rate of dry mass (RGR_DM_) **(C)** of *Potamogeton crispus* and *Vallisneria natans* (*n =* 9 for both species) (The analyses were based on the data in lower layer with a depth of 1.2 m, which is more natural). The red and blue *R^2^ and p* correspond to the red and blue line, respectively.

The relationships between NH_4_ and growth variables for *P. crispus* and *V. natans* in the lower layer (1.2 m), a natural growth depth in the field, are compared in Figure 5. We found that 67% of the relative growth rates of shoot number (RGR_NS_) was higher for *V. natans* than for *P. crispus*, while 78% of the relative growth rates of leaf length (RGR_Length_) and 56% of dry mass (RGR_DM_) of *P. crispus* were systematically higher than those of *V. natans* (Figures 5A–C). The RGR_NS_ of *P. crispus* and of *V. natans* demonstrated no significant relationships with increasing NH_4_ (Figure 5A). RGR_Length_ and RGR_DM_ of *V. natans* both displayed a significant declining trend with NH_4_, while no such trend was found for *P. crispus* (Figures 5B,C).

## Discussion

In an earlier aquarium study by Cao et al. (2009b), an NH_4_ concentration of >1 mg L^-1^ in the water column induced physiological stress on *P. crispus* at low light availability, while neither growth inhibition nor NH_4_ accumulation was observed in the plant tissue of *P. crispus* at normal light availability (Cao et al., 2009b). In addition, high NH_4_ concentrations (>5 mg L^-1^) produced significant acute biochemical changes in *P. crispus* (Cao et al., 2004). Moderate NH_4_ concentrations (0.16–0.25 mg L^-1^) were not directly toxic to *V. natans* (Cao et al., 2011), but an NH_4_ concentration of 1 mg L^-1^ may inhibit the growth and the carbon and nitrogen metabolism of *V. natans* (Cao et al., 2009a). However, in the present study, none of the growth variables of the canopy-forming *P. crispus* (including N_Shoot_, H_Shoot_, and DM_Shoot_) decreased with increasing nitrogen loading, whereas all growth variables decreased with increasing phytoplankton chlorophyll *a*. Five ponds exhibited NH_4_ concentrations (3.1–25.6 mg L^-1^) higher than the above thresholds of 1 and 5 mg L^-1^. This suggests that *P. crispus* was able to overcome the stress induced by high nitrogen concentrations under field conditions. In our previous experiment with the rosette-forming *V. natans*, both leaf length and leaf mass decreased significantly with increasing NH_4_ concentrations (Yu et al., 2017). These results suggest that the canopy-forming *P. crispus* is more tolerant than the rosette-forming *V. natans* when exposed to high NH_4_ concentrations. A potential caveat is that the two experiments were undertaken in two years with different associated pond temperatures and precipitation. In the present experiment with *P. crispus*, the air and water temperature were lower than in the previously published experiment with *V. natans*. In Yu et al. (2017), higher growth rates of *V. natans* were found in the summer-autumn experiment than in the winter-spring experiment, which suggests that *V. natans* growth benefits from higher temperatures, perhaps increasing its tolerance to damage induced by ammonium enrichment. Accordingly, the difference in tolerance of *V. natans* and *P. crispus* would expectedly have been even greater if they had been growing at similar temperatures, further substantiating our hypothesis that canopy-forming submersed macrophytes are more tolerant to NH_4_ stress than rosette-forming species. The amount of precipitation may disturb pond conditions, cause resuspension of the sediment, and create shading effects. When phytoplankton abundance increases, so do the levels of detritus and inorganic suspended matter (see Yu et al., 2018). Although the precipitation in this study was much higher than that in Yu et al. (2017), the average Chl*a*_Phyt_ concentration (41.7 μg L^-1^) did not differ significantly from those in Yu et al. (2017) (33.8 μg L^-1^), indicating that the resuspension of the sediment caused by precipitation might not have had a significant effect on the macrophytes.

Macrophytes may excessively synthesize FAA to avoid NH_4_ toxicity when experiencing high NH_4_ stress. However, this process costs extra energy and consumption of carbohydrates (Rare, 1990; Krupa, 2003), which may reduce the growth of macrophytes, particularly at low light availability (Cao et al., 2009a, 2011; Yuan et al., 2013, 2016). FAA content is therefore commonly used as an indicator of nitrogen stress (Cao et al., 2009b; Zhang et al., 2010; Yuan et al., 2015). In our experiment, FAA tended to increase with increasing NH_4_ concentrations. However, significant positive relationships were recorded between FAA and shoot number (N_Shoot_) (in the middle and lower layers) and shoot height (H_Shoot_) (in middle layer). These positive relationships of FAA with NH_4_ and growth variables, together with weak relationships between NH_4_ and growth variables, suggest that FAA might be a useful indicator of physiological stress by high ammonium but not of macrophyte growth. A similar conclusion was made in our previous pond experiment on *V. natans*, both for the low-growth season (in winter) (Yu et al., 2015) and the high growth season (in summer and autumn) (Yu et al., 2017).

All the growth variables of *P. crispus* declined with increasing concentrations of phytoplankton chlorophyll *a*, suggesting that the decline may mainly be due to shading of phytoplankton, which in our experiment was not significantly related to N loading. Similar results were found for the rosette-forming *V. natans* (Yu et al., 2017). In contrast, all the growth variables of *P. crispus* increased with increasing periphyton chlorophyll *a*. This finding probably reflects the fact that the development of periphyton and macrophytes both rely on underwater light conditions. If light is sufficient, both plant growth metrics and periphyton density will increase. In this experiment, Chl*a*_Peri_, particularly in the middle and lower layers, tended to decrease with enhanced TP. Shading effects by phytoplankton, as suggested by the negative relation between Chl*a*_Peri_ and Chl*a*_Phyt_, were likely promoted by TP. Periphyton on glass may not fully represent those on plants, but measurement of periphyton on glass has its advantages: (1) It does not cause damage to plant tissue and has little impact on plant growth; (2) it is possible to standardize measurement to ensure consistent results. Our results suggest that phytoplankton shading affected both the growth of submerged macrophytes and periphyton negatively.

**FIGURE 6:**
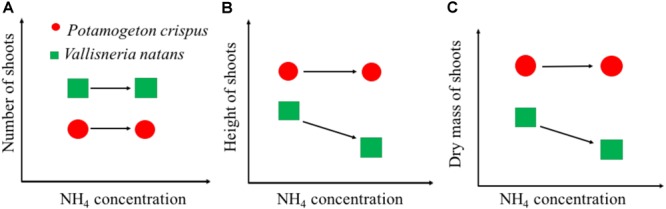
Schematic graphs showing the growth of shoot number **(A)**, shoot height **(B)**, and dry mass **(C)** of canopy-forming *Potamogeton crispus* and rosette-forming *Vallisneria natans* in response to NH4 concentrations from the whole-ecosystem experiments.

Our findings can be summarized with a conceptual model of growth-NH_4_ relationships between canopy-forming *P. crispu*s and rosette-forming *V. natans* exposed to high N stress (Figure 6). Rosette-forming species tend to have a higher number of shoots but lower shoot height and mass than canopy formers, both in the presence and absence of high NH_4_ stress. For canopy-forming species, high NH_4_ stress may not significantly affect any growth variables (Figure 6). By contrast, for the rosette-forming species, high NH_4_ stress may not necessarily affect the shoot number but may lead to a decline in shoot height and mass. The difference in the ability of canopy-forming and rosette-forming species to access underwater light might explain their contrasting tolerance to high NH_4_ stress. In shallow lakes, canopy-forming species are often able to reach the water surface where there is sufficient light to produce carbohydrates and thereby avoid NH_4_ toxicity. Rosette-forming species are often relatively short and therefore relatively prone to low-light conditions. At high N concentrations, rosette-forming species may therefore not produce enough carbohydrates to detoxify NH_4_. Moreover, rosette-forming species become even more affected by high N loading which may cause higher phytoplankton growth or higher periphyton growth as seen in some studies (Özkan et al., 2010; Olsen et al., 2015). Our findings are of practical importance and imply that canopy-forming species may potentially be preferred in the restoration of shallow lakes undergoing high N stress. Moreover, canopy-forming species, if covering the water surface, may block light penetration, limit gas exchange, adversely affect water oxygen concentrations, and probably alter biochemical processes (Turner et al., 2010). However, more tests, especially of different canopy-forming species are needed to draw firm conclusions regarding this study’s conceptual model, so as to cover more species to help better understand the mechanisms underlying macrophyte decline in shallow lakes.

## Author Contributions

QY, H-JW, H-ZW, and EJ designed the research. QY, YL, S-NM, and X-ML carried out the research. QY and H-JW performed the data analyses. QY prepared the original draft of the paper. EJ, CX, and H-JW commented on the various drafts.

## Conflict of Interest Statement

The authors declare that the research was conducted in the absence of any commercial or financial relationships that could be construed as a potential conflict of interest.
