# Supplementary material for: Higher Tolerance of Canopy-Forming Potamogeton crispus Than Rosette-Forming Vallisneria natans to High Nitrogen Concentration as Evidenced From Experiments in 10 Ponds With Contrasting Nitrogen Levels
Source: Front Plant Sci. 2018 Dec 13;9:1845. doi: 10.3389/fpls.2018.01845 (PMC6300520; doi:10.3389/fpls.2018.01845)
Supplement: Supplementary file 1 [file Table_1.DOCX]

Table 1

Environmental conditions for the two experiments.

| Test species and duration | | Rainfall, mm | AT, ℃ | Pond | WT, ℃ | SD, cm | SS, mg L^-1^ |
| --- | --- | --- | --- | --- | --- | --- | --- |
| *Vallisneria natans* (June- September, 2013) | | 1257 | 28.0 | N0.5a | 29.1 | 42.3 | 3.0 |
|  |  |  |  | N0.5b | 29.3 | 38.8 | 11.4 |
|  |  |  |  | N2a | 29.1 | 38.8 |  |
|  |  |  |  | N2b | 29.4 | 41.0 | 20.4 |
|  |  |  |  | N10a | 28.6 | 26.3 | 10.4 |
|  |  |  |  | N10b | 29.5 | 44.5 | 3.4 |
|  |  |  |  | N20a | 29.0 | 34.3 | 7.1 |
|  |  |  |  | N20b | 29.3 | 49.3 | 7.3 |
|  |  |  |  | N100a | 28.9 | 32.8 | 11.7 |
|  |  |  |  | N100b | 29.2 | 56.8 |  |
|  |  |  |  | Average | 29.1 | 52.1 | 9.3 |
| *Potamogeton crispus* (April- June, 2014) | | 2147 | 22.2 | N0.5a | 23.7 | 55.0 | 9.2 |
|  |  |  |  | N0.5b | 23.9 | 65.0 | 16.0 |
|  |  |  |  | N2a | 23.4 | 43.5 | 17.5 |
|  |  |  |  | N2b | 23.7 | 51.5 | 24.8 |
|  |  |  |  | N10a | 23.2 | 25.5 | 81.3 |
|  |  |  |  | N10b | 23.9 | 55.5 | 15.6 |
|  |  |  |  | N20a | 23.7 | 36.5 | 25.9 |
|  |  |  |  | N20b | 23.9 | 62.5 | 14.8 |
|  |  |  |  | N100a | 24.0 | 43.5 | 22.5 |
|  |  |  |  | N100b | 23.6 | 80.5 | 11.6 |
|  |  |  |  | Average | 23.7 | 51.9 | 23.9 |

Note: AT, air temperature; WT, water temperature; SD, Secchi depth, cm; SS, suspended solids, mg L^-1^. Data of rallfall and air temperature were obtained from local weather station. WT and SD were measured once per month. SS was measured once in July 2013 for the *V. Natans* test and in April 2014 for the *P. crispus* test.
